# Outcomes of management of intestinal obstruction at an urban tertiary hospital in sub Saharan Africa: a cross-sectional study

**DOI:** 10.1186/s12893-021-01405-x

**Published:** 2021-11-30

**Authors:** Arlene Muzira Nakanwagi, Stephen C. Kijjambu, Peter Ongom, Tonny Stone Luggya

**Affiliations:** 1 Department of Surgery, College of Health Sciences, Makerere University, Kampala, Uganda; 2 Department of Anaesthesia and Emergency Medicine, College of Health Sciences, Makerere University, Kampala, Uganda

**Keywords:** Acute abdomen, Intestinal obstruction

## Abstract

**Background:**

Intestinal obstruction (IO) is a common cause of acute abdomen globally, it remains challenging as it increases surgical financial expenditure while also causing major morbidity. Clinically it presents with nausea, vomiting, colicky abdominal pain and cessation of bowel movements or passage flatus and stool. Diagnosis, especially in resource limited settings, can be clinical but is usually confirmed radiologically. We studied the current diagnosis, management and outcomes of IO in Mulago Hospital.

**Materials and methods:**

This was a prospective study done at all the surgical units of Mulago from January to May 2014 to assess general diagnosis and management of IO. Ethical approval was got in line with Helsinki declaration, we used pretested and validated questionnaires to collect data. Informed consent was got with eligible and consenting/assenting patients that fitted the inclusion criteria of age and presenting with suspected intestinal obstruction. Uni-variate and bi-variate variables analysis was done plus measures of association.

**Results:**

We enrolled 135 patients, excluded 25 and recruited 110 patient. We had more males than females i.e. 71.8% males and 28.2% females. Colicky abdominal pain, abdominal distension, and vomiting were commonest symptoms, then abdominal distension, increased bowel sounds and abdominal tenderness were the commonest signs. Most patients’ (51%) were diagnosed radiologically with a lesser number clinically diagnosed. “Dilated bowel loops” was the commonest radiological sign. Surgery was the main stay of management at 72.7% while 27.3% were conservatively managed. Postoperatively the bowels opened averagely on the 3rd post-operative day (POD) with return of bowel sounds occurring on 5th POD. Most discharges (73%) occurred by the 7th POD. Unfavourable outcomes were prolonged hospital stay followed by wound sepsis (surgical site infection) and then Mortality.

**Conclusion:**

This study noted that In Mulago we mostly diagnosed patients radiologically with most surgically managed and which is similar to regional practices. Postoperatively bowel opening happening on third POD with return of bowel sounds on fifth POD. Prolonged hospital stay followed by wound sepsis and then mortality were commonest unfavorable management outcomes.

## Background

Intestinal obstruction (IO) refers to the forward flow interruption of intestinal contents occurring at any point from mouth to anal canal, usually presenting with clinical symptoms that vary based on the level of obstruction [1]. It’s the leading cause of acute abdomen globally and is characterized by abdominal pain, which in 2006 was reported as the USA’s commonest reason for a visit to the Emergency Department [ED]. IO accounts for 8 million (7%) of the 119 million visits [2] and is the most frequently encountered emergency leading to abdominal surgery [3, 4]. It still remains a challenge as it causes morbidity and increased financial expenditure worldwide [5]. In the USA IO accounts for about 30,000 deaths plus $3 billion per year in direct medical costs which are responsible for approximately 15% of hospital admissions for acute abdominal pain with approximately 20% of these cases needing acute surgical care [6, 7].

Clinical presentation generally includes nausea, vomiting, colicky abdominal pain and cessation of bowel movements or passage flatus and stool, although the severity of these clinical symptoms varies based on the acuity and anatomic level of obstruction [1, 7]. With a high index of suspicion diagnosis is usually confirmed radiologically with X-ray and ultrasound although, non-contrast computed tomography (CT) [1] is the main modality of diagnosis in advanced centers. Management of uncomplicated obstructions, commonly due to adhesive intestinal obstruction, can be conservative by fluid resuscitation to correct the metabolic derangements, intestinal decompression, and bowel rest. If there’s evidence of vascular compromise or perforation then management is by surgical intervention [7]. Regional studies at non-governmental hospitals of Uganda documented its incidence and management [8, 9]. However at Mulago National Referral and Teaching Hospital (MNRTH), which is the nation’s tertiary health care institution offering free services to patients, has anecdotal data from a  study done in 1961 [9].  Also  raw admission data from MNRTH ED shows  an increasing surgical burden of averagely 30 patients monthly with suspected diagnosis of IO [10].

We thus set out to study the current diagnosis, management and outcomes of IO in MNRTH especially due to the paucity of data to guide current policy on decision making process, resuscitation measures, timing of surgery and choice of surgical procedure in our setting.

## Materials and methods

### Study setting

MNRTH is located in Kampala the Capital City of Uganda and is a 1500 bed tertiary hospital caring for approximately 140,000 patients annually with an annual average of 48,000 patients transitioning through the ED [11]. It has three surgical wards, with an A&E that triages patients into elective or emergency cases done in casualty theatre that records about 31 operations monthly for the relief of IO [10]. Also A&E records show that on average about 7 to 10 patients’ with IO are seen per week by the Senior House Officer’s (SHO’s).

### Ethical approval and recruitment

Ethical approval was obtained from the Department of Surgery, Makerere University School of Medicine Research an Ethics Committee (SOMREC) and MNRTH IRB to conduct a Prospective Descriptive Study in all the surgical units of MNRTH. It was a prospective descriptive cross sectional study of 110 patients selected by the convenient sampling method. Our study population was both male and female patients of all ages that presented to the surgical units with suspected IO.

Consent was got from all patients and for subjects below 18 years, a parent, legal guardian or Next of Kin gave consent on their behalf. Eligible and consenting patients were recruited with details of their demographics, symptoms and signs recorded. Clinically Bowel sounds were assed as hyperactive, hypo active or absent. Outcome measures were assessing diagnosis, resuscitation, investigations and treatments then monitored till discharge or death. The favourable outcomes was the relief of IO with resolution of symptoms and patient discharged in perfect health. The unfavorable outcomes were those associated with increased patient morbidity i.e. secondary complications and length of stay plus mortality. The causes of IO, 7 days post-operative/post admission management outcomes including deaths or up to discharge, whichever came first, were recorded.

#### Study periods

The study was conducted from January to May 2014.

### Study variables

The main variables of interest were clinical examination, radiologic findings, and management options focusing on favorable and unfavorable outcomes in the Post-Operative Day (POD) window.

### Sample size

Using Kish-Lesley sample size formula for proportion with 95% confidence interval and 30% loss to follow up our sample size was 94 patients, we however recruited 110 patients.

### Data management and analysis

A structured pretested and validated questionnaire was used to collect data. The data was cleaned, backed up and later analyzed in STATA version10.1. Study statistics were reported using proportions, means, medians and inter-quartile ranges. Uni-variate and bi-variate analysis of the variables plus measurements of associations were done using Pearson Chi-*χ*^2^ values, *p* values and logistic regression. Presentation of data was by bar graphs, pie charts, Box-plots and Tables.

## Results

We recruited 110 patients that met the inclusion criteria plus outcomes of interest i.e. investigations suggestive of obstruction, or confirmation of diagnosis at laparotomy with favorable and unfavorable outcomes. Demographically there were more males (71.8%) than females (28.2%) in a ratio of 2.6:1. The 3 commonest symptoms reported by the participants were; colicky abdominal pain, abdominal distension, and vomiting. The 3 commonest examination signs were; abdominal distension, increased bowel sounds and abdominal tenderness. Majority of the acute abdomen patients were diagnosed radiologically (51%) with clinical diagnosis accounting for 48.2%. The commonest etiological factor for IO was obstructed hernias, then intestinal volvulus, adhesions and tumors in third place. However of the 80 cases (72.7%) were eventually operated and we noted that a higher majority (58.8%) were clinically diagnosed (See Table 1). Of the eighty patients operated, resection and anastomosis was the commonest procedure (35.0%) followed by hernia repair at 23.8% and then Simple colostomy at 15% and laparotomy with colostomy as the least common procedure.

**Table 1:** Mode of diagnosis by choice of management

Characteristic	Overall 110 (100%)	Non-operative 30 (27.3%)	Operative 80 (72.7%)	p-value (Non-operative vs. operative)
Diagnosis n (%)				0.001*
Radiological	57 (51.8)	24 (80.0)	34 (41.3)	
Clinical	53 (48.2)	06 (20.0)	46 (58.8)	

“Dilated bowel loops” was the most frequent sign found on both imaging modalities. Multiple air fluid levels were observed in 12.7% of the radiographs and faecal impaction was noted in 5.5% of the X-rays. On Sonography, reversed peristalsis was noted in 10% of the cases. The other signs were seen 5.5% of the cases. Ultra sound Sonography exerted more influence on the choice of management when compared to plain radiography. (p = 0.001 vs. p = 0.013). *Shown in* Table 2

**Table 2:** Radiological signs by choice of management

Radiological signs	Overall 33 (30%)	Non-operative 30 (27.3)	Operative 80 (72.7)	p value
Plain X-ray	32 (29.1)	14 (46.7)	18 (22.5)	0.013
Dilated bowel loops	29 (26.4)	12 (40.0)	17 (21.3)	< 0.001
Multiple air levels	14 (12.7)	01 (3.3)	13 (16.3)	0.07
Fecal impaction	06 (5.5)	06 (20.0)	00 (0.0)	0.047
Sonographic findings	33 (30.0)	16 (53.3)	17 (21.3)	0.001
Dilated bowel loops	29 (26.4)	12 (40.0)	17 (21.3)	0.047
Reversed/no peristalsis	11 (10.0)	05 (16.7)	06 (07.5)	0.154
Doughnut sign	06 (05.5)	02 (06.7)	04 (05.0)	0.732
Abdominal masses	06 (05.5)	05 (16.7)	01 (01.3)	0.002
Peritonitis	06 (05.5)	00 (00.0)	06 (05.5)	0.161

Outcomes were categorized as favourable or unfavorable with the favourable outcomes being: return of bowel sounds, opening of bowel, NG-tube removal, drain removal and discharge by the 7th post-operative or post admission day (for non-operative management). The unfavorable outcomes included: wound sepsis, systemic sepsis, anastomotic leak, anemia, chest infections, prolonged hospital stay and death.

**Fig. 1:**
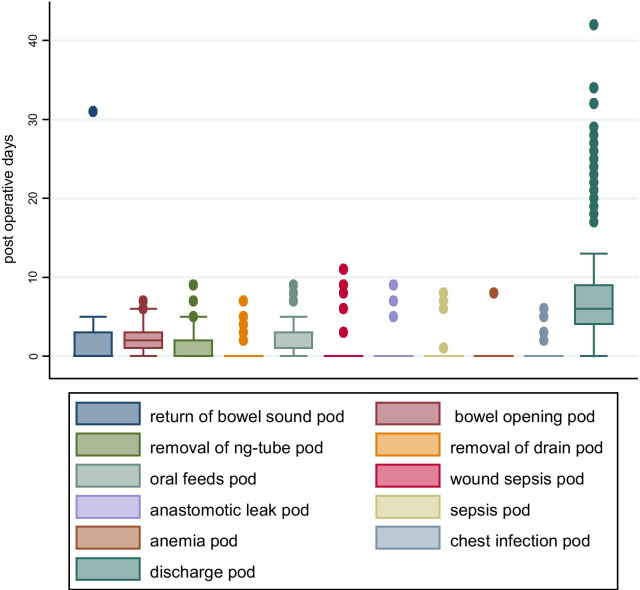
Box plot showing post-management events by post-operative/ admission day

For the favourable outcomes; opening of bowels on average occurred on the 3rd POD, bowel sounds returned by 5th POD and majority (73%) of the patients were discharged by the 7th POD which was the average hospital stay (Fig. 1).

**Fig. 2:**
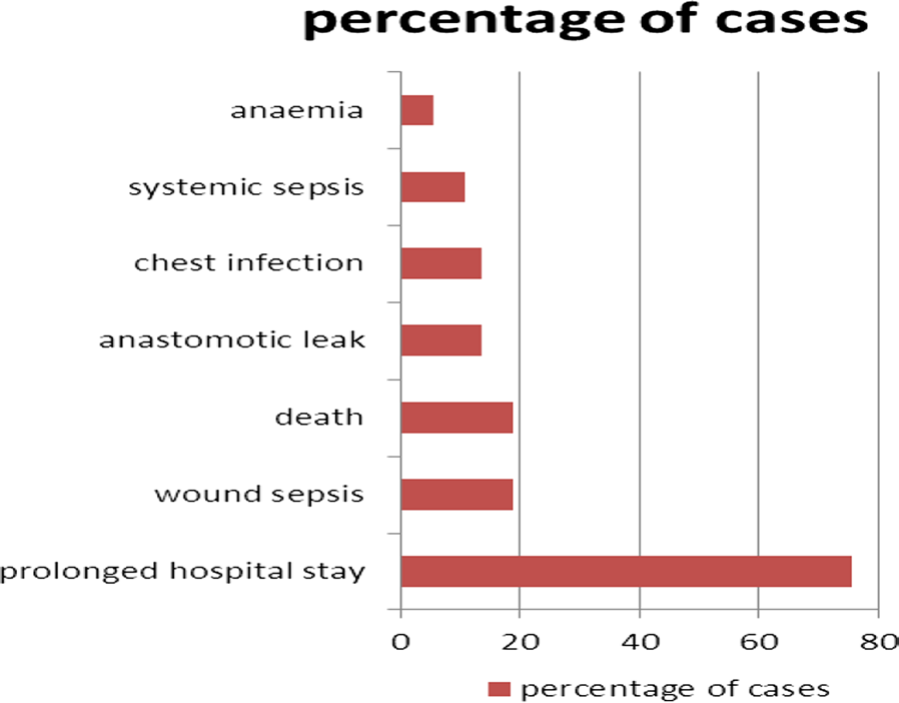
Bar graph showing percentage distribution of unfavourable outcomes

The commonest unfavourable management outcome noted was prolonged hospital stay (73 patients) followed by wound sepsis (Surgical Site Infection) and Mortality (Fig. 2).

**Fig. 3:**
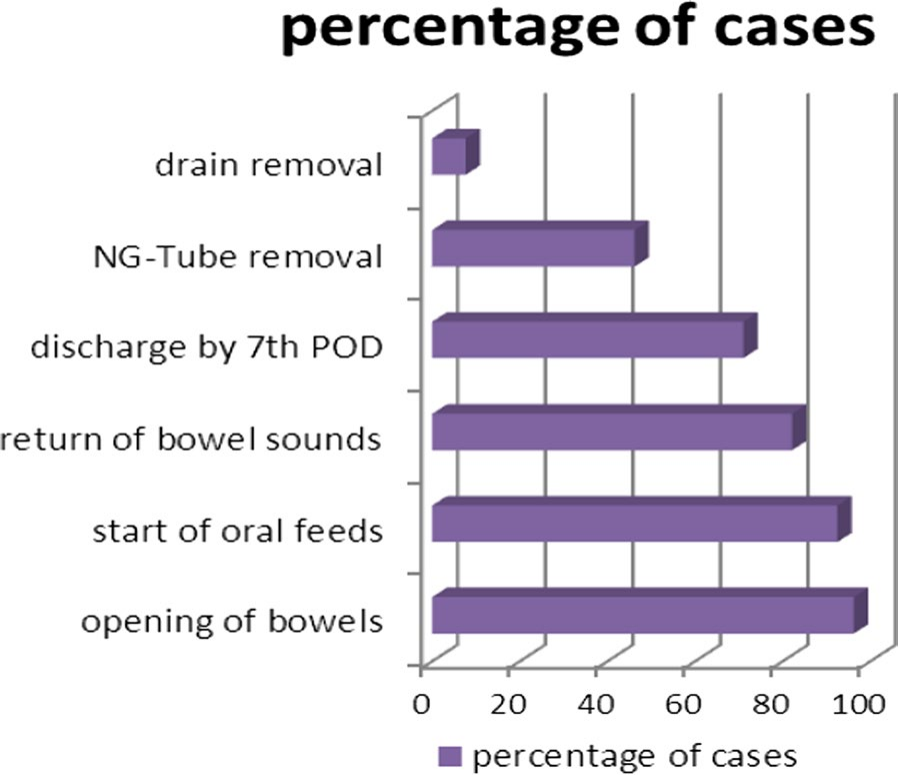
Bar graph showing distribution of favourable outcomes by frequency

Factors that were persistently associated with favorable outcomes, in the Uni-variate model, were fever (RR = 0.19, *p* = 0.09), Increased bowel sounds (RR = 4.3, *p* = 0.001). Absent bowel sounds and abdominal tenderness (*p* = 0.001 and *p* = 0.014) showed association with favorable outcomes at lower odds. Small bowel obstruction (SBO) is 3.17 likely to be associated with favorable outcomes (*p* = 0.013). See Fig. 3 and Table 3*.*

**Table 3:** Displays modified Cox regression analysis used to assess the significant factors associated with outcome (p < 0.05)

Predictor of outcome	Relative-risk	Confidence interval	p-value
Symptom duration			
24–48 h	1.83	0.289–12.07	0.528
48–72 h	0.67	0.149–2.970	0.596
> 72 h at home	0.52	0.132–2.012	0.340
> 72 h in health center	0.29	0.075–1.112	0.075
Operative vs. non-operative management	0.21	0.067–0.650	0.007
Symptoms of IO			
Abdominal pain	0.93	0.382–2.256	0.870
Fever	0.19	0.540–0.665	0.009
Vomiting	0.59	0.220–1.181	0.116
Relative constipation	0.67	0.297–1.533	0.348
Absolute constipation	1.33	0.537–3.280	0.540
Signs			
Abdominal distension	0.56	0.202–1.563	0.270
Absent bowel sounds	0.24	0.968–0.522	0.001
Increased bowel sounds	4.28	1.855–9.861	0.001
Abdominal tenderness	0.36	0.162–0.817	0.014
Percussion tenderness	1.61	0.162–16.02	0.685
Sonographic signs of IO			
Reversed peristalsis	0.398	0.113–1.402	0.152
Peritonitis	0.333	0.053–2.088	0.241
Abdominal masses	1.058	0.185–6.061	0.969
Clinical vs. radiological diagnosis	0.762	0.346–1.674	0498
Level of obstruction (SBO vs. LBO)	3.17	1.280–7.861	0.013
Bowel status			
Strangulation	0.50	0.155–1.611	0.245
Gangrene	0.20	0.059–0.723	0.014
Perforation	0.44	0.090–2.189	0.319
peritonitis	0.25	0.508–1.231	0.088
Urethral catheterization	0.22	0.617–0.789	0.020

These were selected for this further analysis

The choice of management, i.e. non-operative versus operative, greatly influenced outcome with most conservatively managed patients having favourable outcomes.

The results in Table 4 below show the recomputed odds ratio in a multivariate regression and unlike in the binary logistic regression, none of the factors independently predict the outcome, implying that they most likely act in association or in combination with one another, as their relative risk changes and the p value becomes statistically insignificant.

**Table 4:** Multi-variate analysis of statistically significant variables in the uni-variate regression

Predictor of outcome	Relative- Risk	Confidence interval	*p* value
Choice of management (operative)	0.39	0.111–1.342	0.134
Level of obstruction	1.85	0.622–5.592	0.266
Signs of IO			
Fever	0.28	0.599–1.335	0.111
Increased bowel sounds	1.35	0.176–10.34	0.770
Absent bowel sounds	0.48	0.058–3.946	0.493
Abdominal tenderness	0.72	0.255–2.035	0.530
Percussion tenderness	6.99	0.545–89.81	0.140

## Discussion

We studied the diagnosis, management and early outcomes IO in MNRTH. Baseline demographics showed more males (71.8%) than females (28.2%) were affected with majority radiologically diagnosed (51.8%) which was significant in comparison to other African studies [12–14]. The diagnostic value of radiographs was depicted in the strong statistical correlation between imaging and choice of management (Table 2), with X-rays having a *p* = 0.013, while Sonography had a *p* = 0.001. Studies have shown that there is also increasing reliance on radiological investigations when the immediate choice of management is conservative rather than operative as most cases resolve spontaneously [15]. Etiology of IO in this study showed 69.6% of the obstruction hernias were in adults and 7 (30.4%) in children.

Management of IO in our resource limited environment at MNRTH was majorly (72.7%) operative, with resection and anastomosis (35.0%) as the commonest procedure followed by hernia repair (23.8%), then Simple colostomy (15%) and laparotomy with colostomy as the least common procedure. Seventy three (66.4%) cases had favourable outcomes while 37 (33.6%) had associated morbidity and mortality. Prolonged hospital stay (30.4%) which was the commonest adverse outcome was similar to regional study findings [8, 9, 16–18].

Prolonged hospital stay was associated with resection and anastomosis, followed by ileostomy and we posit it was due to “tumor” diagnosis for which these patients had await pathological diagnosis, prepped for staging investigations or radiotherapy. We had low mortality compared to other regional studies that had rates of 12.9%, 19.7% and 20% [9, 17, 19, 20].

A critical aspect of management of IO is to determine whether to operate or manage conservatively [21]. We had low rates of conservative management (27.3%) in comparison to high resourced centers like USA (73%) which we postulate that could probably be attributed to the lack of alternative methods of non-operative management such as Gastrograffin use in adhesions, hydrostatic or pneumatic reduction of intussusception, as well as the lack of investigative capacity to confirm or rule out bowel ischemia in our setting [16, 22–24]. Also since MNRTH is a tertiary center, attending to cases from primary and tertiary centers hence could have affected our conservative management options, with serious and advanced in progression cases presenting.

This study had some draw backs like the recall bias since some patient data was from records plus postoperative interviews by the operating surgeon or SHO which may have affected some study variables collected.

## Conclusion

This study noted that In Mulago we mostly diagnosed patients radiologically with most surgically managed and which is similar to regional practices. Postoperatively bowel opening happening on third POD with return of bowel sounds on fifth POD. Prolonged hospital stay followed by wound sepsis and then mortality were commonest unfavorable management outcomes.

## Data Availability

The data supporting the findings of this study, other than that is available within the article*,* are available upon request from the corresponding author.

## Abbreviations

MNRTH: Mulago National Referral and Teaching Hospital
IO: Intestinal obstruction
SBO: Small bowel obstruction
LBO: Large bowel obstruction
POD: Post-operative day
PAD: Post admission day
SHO’s: Senior House Officers
